# Iatrogenic combined common iliac and lateral sacral artery perforation during coronary angiography: A case report and review of literature

**DOI:** 10.1002/ccr3.8903

**Published:** 2024-05-20

**Authors:** Maryam Mehrpooya, Massoud Ghasemi, Pouya Ebrahimi, Homa Taheri, Parnian Soltani

**Affiliations:** ^1^ Department of Cardiology, Imam Khomeini Hospital Tehran University of Medical Sciences Tehran Iran; ^2^ Department of Interventional Cardiology Research Center of Endovascular Intervention, Imam Khomeini Hospital Complex Tehran Iran; ^3^ Tehran Heart Center Research and Development Center, Tehran University of Medical Sciences Tehran Iran; ^4^ Cardiology Department Cedars‐Sinai Hospital California USA

**Keywords:** cardiology, coronary angiography, coronary artery disease, endovascular procedure, iatrogenic disease, iliac artery

## Abstract

**Key Clinical Message:**

Arterial rupture is one of the rare but known and devastating complications of the angiogram, which can ultimately lead to loss of limb and life. Therefore, it is recommended that this complication be included in the consent form and that the operator and the logistics team be prepared for this scenario. Moreover, categorizing the patients based on risk factors to be more cautious during the procedure for high‐risk patients can be considered a reasonable strategy.

**Abstract:**

One of the rare but lethal complications of femoral artery catheterization for coronary angiography is arterial rupture, which can cause a range of negligible to massive retroperitoneal hemorrhage. This case presents a woman with unstable angina who underwent coronary catheterization. After arterial sheath placement, extravasation of blood from the right common iliac and lateral sacral arteries was seen, a diagnosis that has been reported rarely before. The bleeding was controlled with balloon inflation in the lateral sacral artery and a stent graft implantation in the right common iliac artery. The patient remained asymptomatic during the procedure and the short‐ and long‐term follow‐up. Interventional cardiologists and radiologists who access the femoral artery for any procedure should be aware of this possible event. Sometimes, this situation manifests with nonspecific symptoms such as weakness, lethargy, and pallor. Moreover, more logistical preparation and training are needed to overcome these unexpected conditions.

## 1 INTRODUCTION

Percutaneous interventions have gradually replaced open and complicated coronary and peripheral artery surgeries in the recent era.^1^ Catheters and guidewires are the mainstays of these procedures that help navigate blood vessels. The significant benefits of these procedures include less than 1% of major complications and shorter admission times for patients in the hospital.^2^ On the other hand, a greater desire to use these methods has caused a higher prevalence of complications of these interventions. One of the rarest and most lethal of these complications is perforation of the arteries and massive hemorrhage.^3^ In this case, a woman who presented with unstable angina complicated by perforation of the right common iliac and lateral sacral arteries during coronary angiography is explained. Furthermore, the place of perforation, the final dissection at the end of the procedure, and the clinical presentation of the patient add to the uniqueness of this case.

## 2 CASE PRESENTATION

### 2.1 Case history and examination

The patient was a 70‐year‐old female with diabetes mellitus, and without any other past medical history, presented to the emergency room due to chest pain that started a few hours ago. The pain was intermittent, described as retrosternal, moderate, worsening with walking and exertion, and improving with rest. There was no radiation of the pain to the arms or jaw. Her previous myocardial perfusion scan showed evidence of mild ischemia in the anterior wall. In the physical exam, her vital signs were stable and normal except for her blood pressure, 150/80 mmHg.

### 2.2 Methods

**FIGURE 1:**
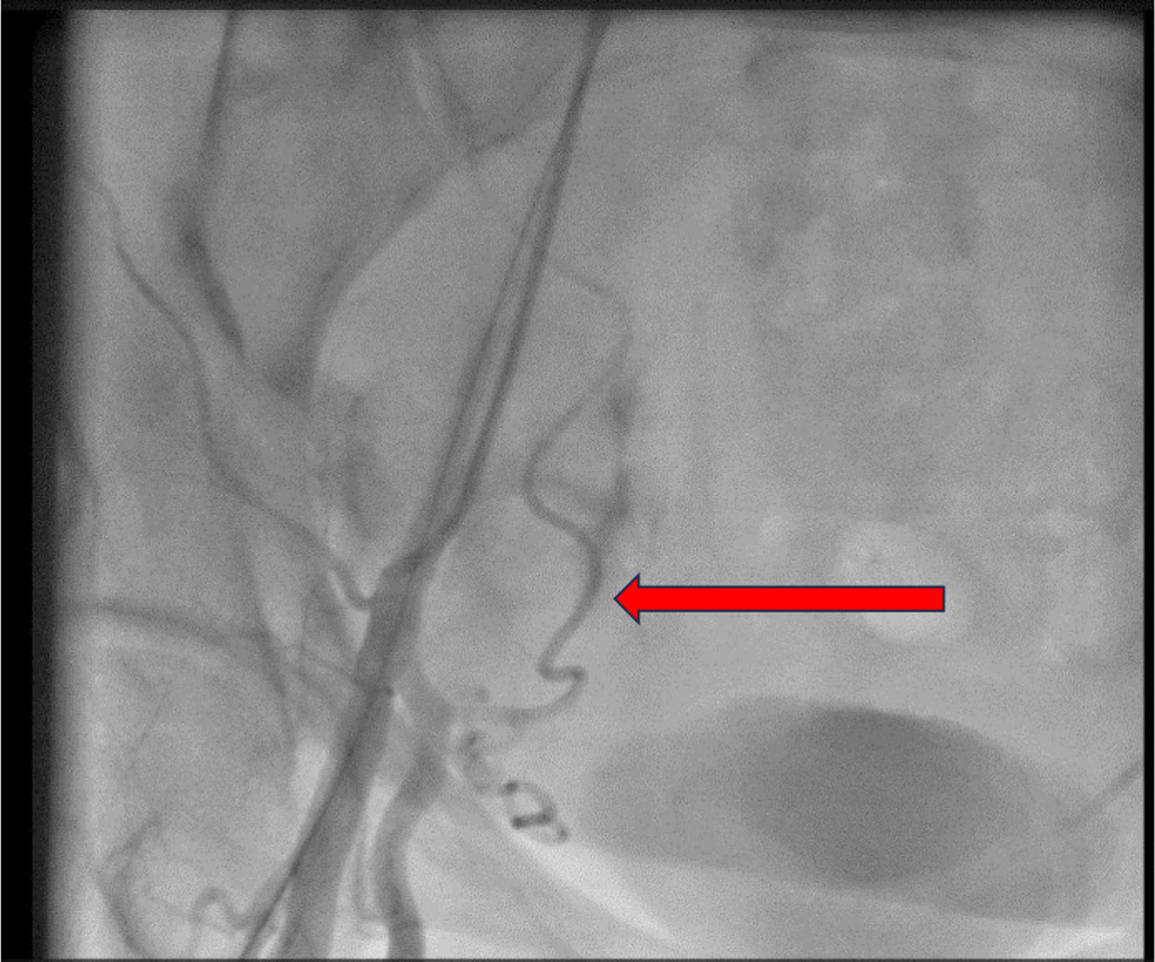
Contrast media extravasation from the lateral sacral artery.

**FIGURE 2:**
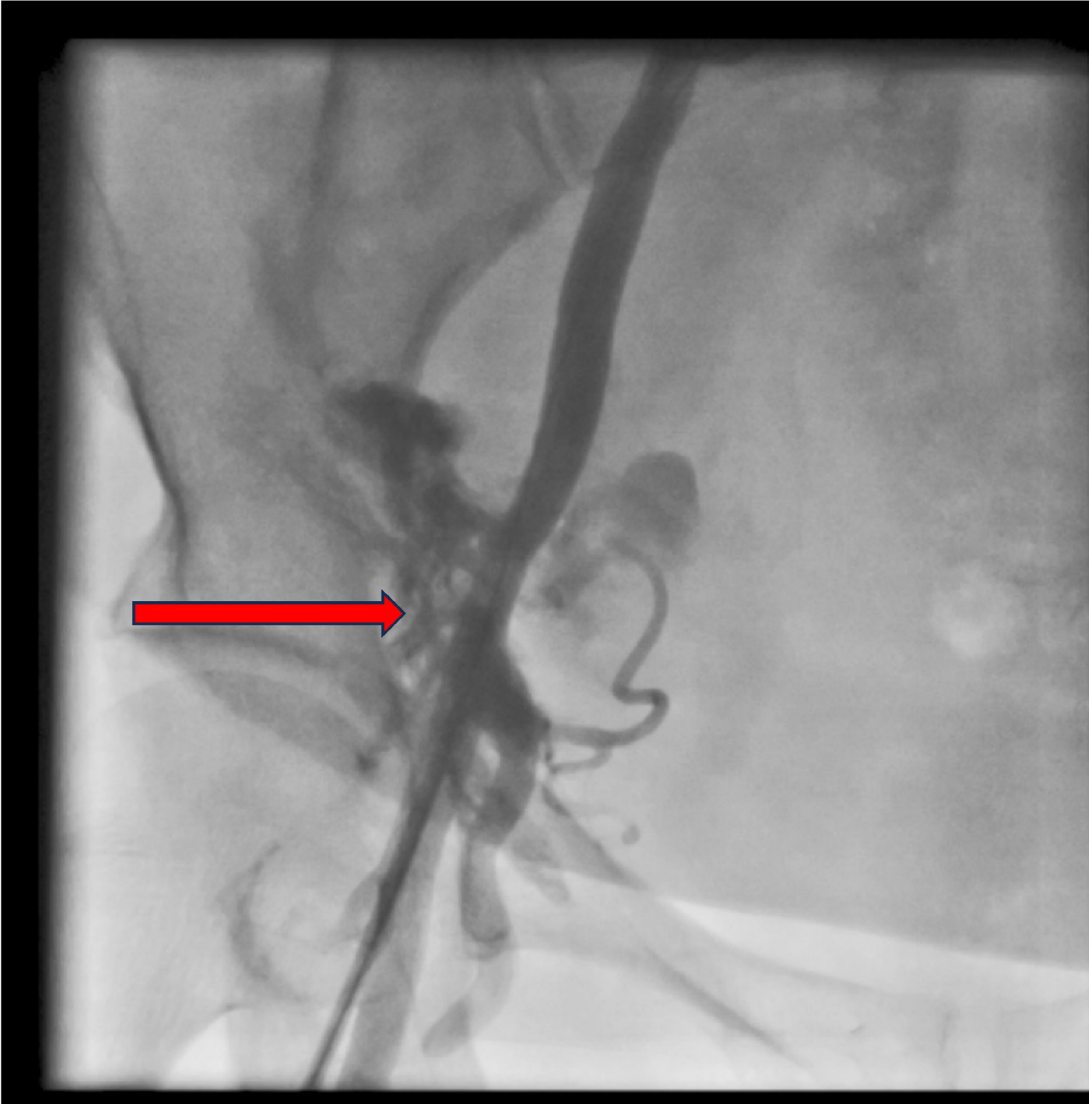
Perforation of the right common iliac artery (contrast media extravasation).

The patient was admitted to the emergency room. Her electrocardiogram showed normal sinus rhythm and no axis deviation, along with an inversion of T wave in leads one and AVL and also in V1–V4. In her bedside echocardiogram, abnormal findings were an Ejection Fraction of 45%, regional wall motion abnormalities, and mild mitral regurgitation. Therefore, considering the risk of acute coronary syndrome and the progression of the disease, we admitted the patient for further evaluation. The medical treatment started with aspirin, clopidogrel, atorvastatin, metoprolol, captopril, and intravenous nitroglycerin. Although all of her laboratory data were unremarkable, her first‐time troponin was borderline. The patient was admitted to the coronary care unit. We decided to do an urgent coronary artery angiography, suspecting the risk of occlusion of the coronary arteries. The patient was prepared for coronary angiography and transferred to the catheterization laboratory. Due to impaired Allen's test for radial access, right femoral artery access was provided. Moments later, the patient felt extremely nauseous and became hypotensive. By injecting it into the arterial sheath, we noticed contrast media extravasation from the lateral sacral artery (Figure 1) and the right common iliac artery (Figure 2), which should be sealed promptly.

**FIGURE 3:**
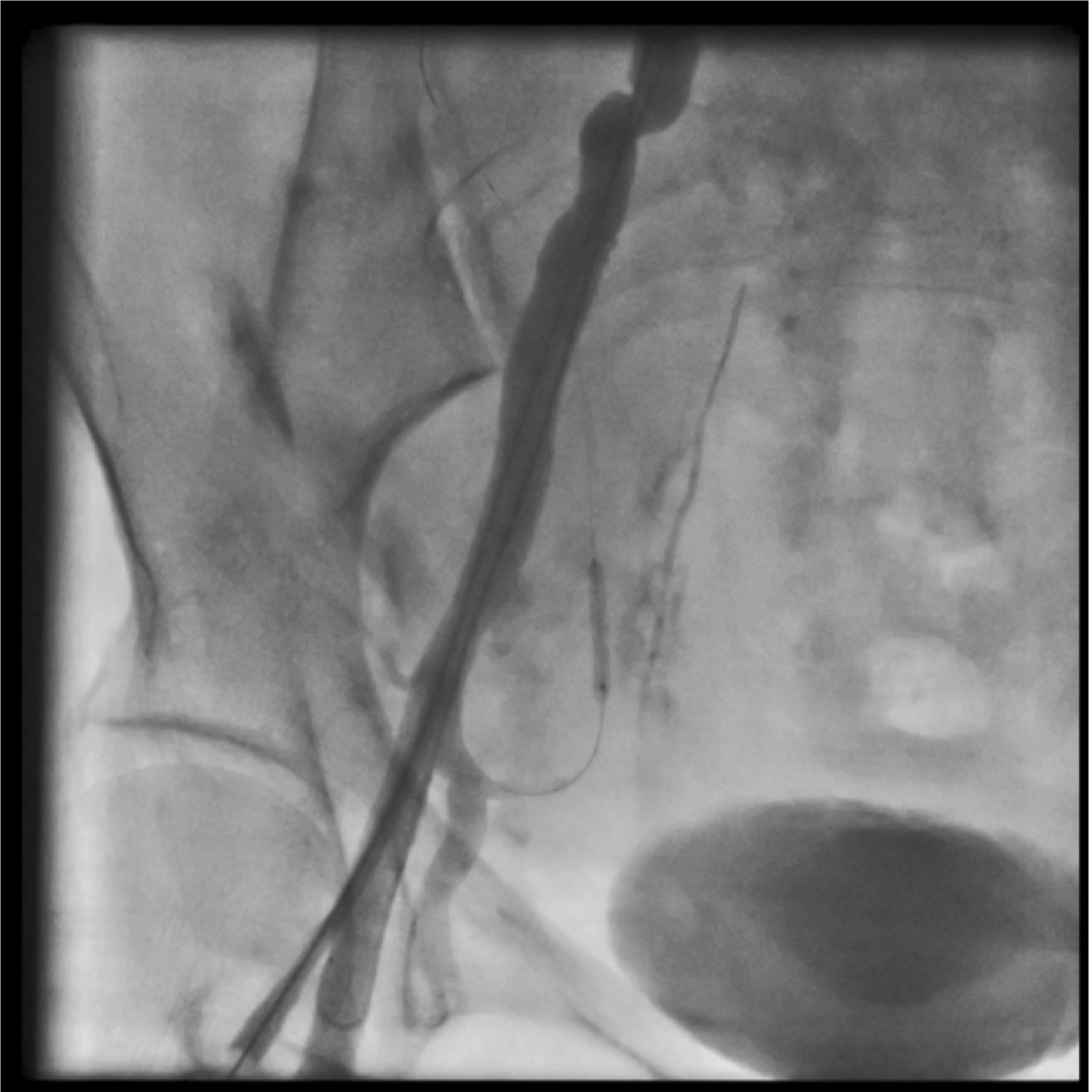
Balloon inflation in the lateral sacral artery.

**FIGURE 4:**
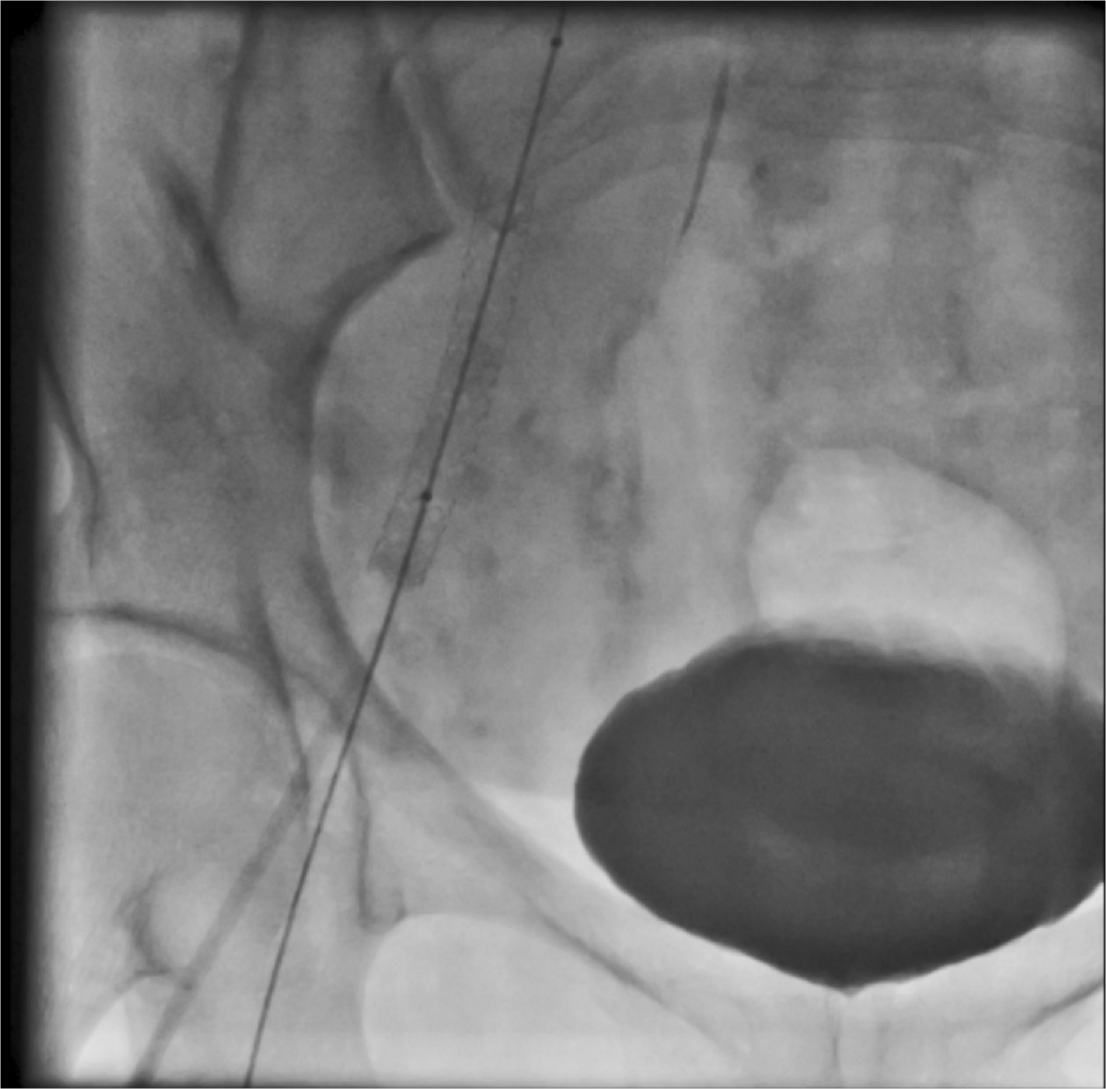
Implantation of the stent graft in the right common iliac artery.

**FIGURE 5:**
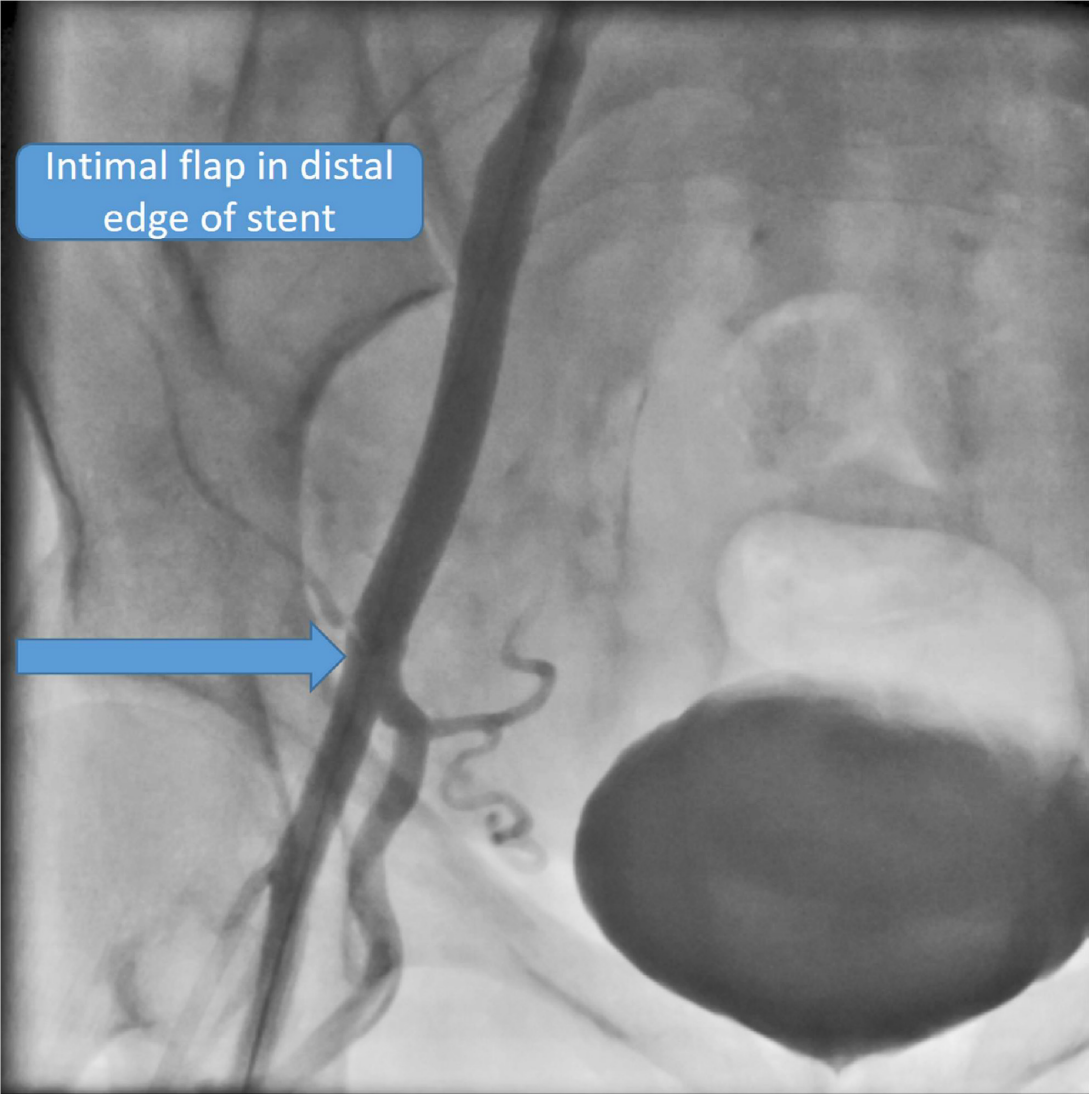
Intimal flap in the distal edge of the stent.

**FIGURE 6:**
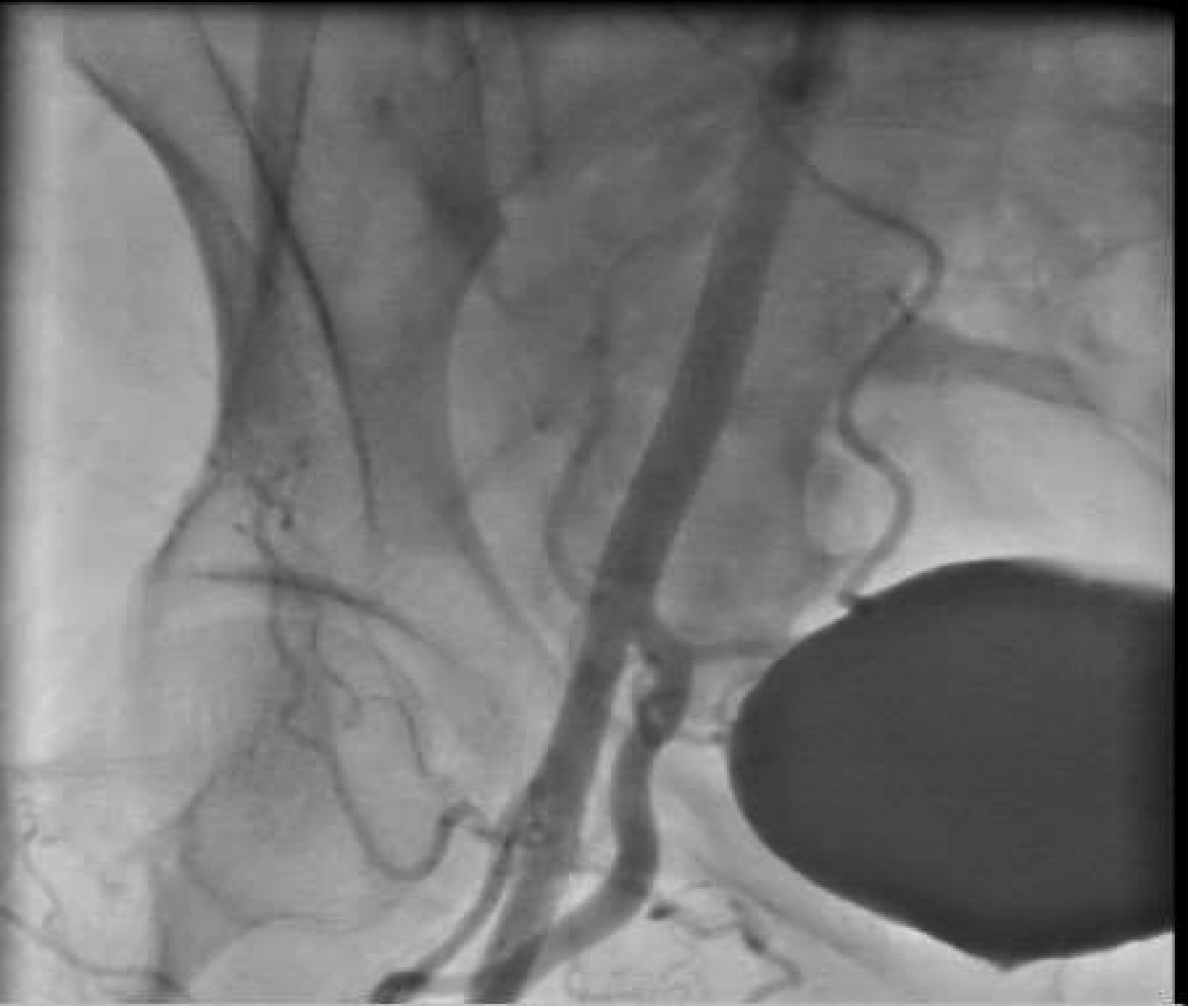
Normal flow without any extravasation.

First, arterial access was provided from the opposite side. Wire 0.014 inch was passed through the lateral sacral artery and inflated with a coronary balloon 1.5 × 15 (Figure 3).Then we stented the main body of the right common iliac artery with a stent graft (Be Graft Bently 8 × 57) (Figure 4) However, surprisingly, a small linear dissection (small flap) was seen in the distal edge of the stent (Figure 5). According to consultation with other interventional colleagues, it was decided that no other aggressive action should be taken. The final injection of the dye into the right common iliac artery showed normal flow without any extravasation in addition to sealing of the dissection site (Figure 6).

### 2.3 Outcome and follow‐ups

The patient's condition gradually improved, and no significant abnormality was observed in the coronary angiography. She was transferred to the coronary care unit. Her hospital course was uneventful, and she was discharged 3 days after the procedure without any medical problems. She received 6‐month Dual Anti‐Platelet Therapy (ASA + Plavix). Her 1‐year follow‐up was excellent, and she did not have any cardiac or peripheral symptoms or events.

## 3 DISCUSSION

Transfemoral arterial access complications consist of a wide range of severity and symptoms. They can be as negligible as minor local hemorrhage, expanding groin hematoma, arteriovenous fistula and pseudoaneurysm, and massive retroperitoneal hemorrhage.^4^ Although it happens rarely, a severe form of this perforation, which can result in massive retroperitoneal hemorrhage, can cause rapid deterioration, collapse, and the death of a patient in a short period.^5^ The incidence of retroperitoneal hematoma formation in interventional endovascular procedures is less than 1%, but the mortality risk of incidents can be as high as 4%–12%.^6^ Therefore, the necessary setting should be available, and the interventional specialists should be prepared to tackle this problem in all transfemoral catheterizations.

Several risk factors have been identified for the perforation of vessels during these procedures, including all causes of increased stiffness of the medial artery (such as uncontrolled diabetes mellitus, hypertension, chronic kidney disease, and older age).^7^ Abnormal anatomical features and tortuosity, more prominently in cases of using straight‐tipped wires and use of large sheaths (mainly for transcatheter aortic valve implantation and endovascular abdominal aortic repair) are risk factors that cause a higher risk of vascular perforation.^6^, ^8^ Another known risk factor for vessel perforation is using a catheter and leading it forward without wire. Furthermore, higher angioplasty pressure, longer time of the procedure, use of anticoagulants and thrombolytics, and long‐term use of steroids are other modifiable causes of vessel perforation during these procedures.^9^ Table 1 shows the modifiable and non‐modifiable factors. In the presented case, it seems that patients' risk factors such as old age, diabetes mellitus, and vascular structure were the non‐modifiable contributing to this event. Moreover, despite a highly attentive approach and paying attention to avoid any redundant guidewire manipulation, we suspect this reason might have also contributed to the perforation. However, more evaluation of the procedure by the recorded data showed no redundant manipulation.

**TABLE 1:** Modifiable and non‐modifiable risk factors for iatrogenic vascular complications.^9^

Modifiable	Non‐modifiable
Large sheath size	Vascular calcification
Redundant guidewire manipulation	Vascular tortuosity
High angioplasty pressures	High‐grade stenosis
Procedure time	Older age
Anticoagulant and thrombolytics	Diabetes mellitus
Long‐term steroid therapy	Chronic kidney disease

The EUROSTAR registry and several other studies have suggested performing maneuvers for more convenient access.^10^ One of the main causes of vessel perforation during endovascular procedures is the anatomy of the iliac arteries. These risk factors might even lead to procedure failure.^11^ Another study showed women, in comparison with men, have had narrower iliac arteries, higher calcification scores, and lower tortuosity indexes.^12^ Therefore, gender‐associated factors can cause an increased or decreased risk of vessel perforation. Moreover, there is no common consensus regarding the reliability of Alle's test in predicting the risk of ischemia by radial access.^13^ However, a study performed in 2007 showed this test has 73.2% sensitivity and 97.1% specificity.^14^ On the other hand, a meta‐analysis performed in 2017 showed a sensitivity of 93% and an interobserver agreement rate of only 71.5%, which questioned the reliability of the test.^15^

The trend of treatment for perioperative iliac rupture has changed toward endovascular techniques instead of open repair. This method's benefits are avoiding the necessity of general anesthesia, wound complications, and a shorter recovery time and hospital stay. However, in some cases, such as compartment syndrome, open surgery is inevitable.^11^ Higher mortality and morbidity of iliac artery rupture can be due to significant hypotension and resulting multiorgan failure, spinal ischemia, and other fatal consequences.^16^, ^17^

To manage these perforations, as soon as a vessel perforation is identified, rapid usage of balloon tamponade, along with the rapid reversal of anticoagulants and antiplatelets, is necessary. At the same time, intravenous fluids and blood products should be utilized for hemodynamic stabilization in these patients. Therapeutic options such as vascular surgery consultation and the use of covered stents should also be considered along with conservative treatment.

Endovascular interventions are preferred due to higher rates of morbidity and mortality associated with vascular surgeries^9^; great advancement has especially been seen by using covered stents instead of open surgical techniques.^18^ In Table 2, we explain cases of external iliac, common iliac, and femoral artery perforation that have been managed efficiently with the covered stents.^6^, ^19^, ^20^

**TABLE 2:** Similar cases of endovascular repair.

Study and authors	Device	Treatment method and device	Age and sex	Type of surgery	Vascular injury
1. Muhammad Umer Awan et al.^6^	14‐in. impella sheath	A stent deployed to the left external iliac artery/successful perforation sealing/required 4 units of packed cell transfusion/complicated by transient AKI/discharged 10 days later without any medical problem	70‐year‐old male	Percutaneous coronary intervention (PCI) of LAD	Left common iliac artery
2. U. Nyman et al.^19^	A guidewire followed by a 7F introducer+ One 8 × 70 mm and one 7 × 90 mm self‐expandable nitinol stent (Memotherm, Angiomed/Bard, Karlsruhe, Germany)	Reinsertion and inflation of the angioplasty balloon./the right CIA was then occluded using a balloon catheter, removal of angioplasty catheter/deploying self‐expandable Stent covered with polyester fabric. Thrombosis of the stented vessels/heparin & thrombectomy/final full recovery	52‐year‐old woman	Percutaneous recanalization of chronic occlusion of the right common (CIA) and external iliac artery (EIA)	External iliac artery
3. U. Nyman et al.^19^	Using a 5F catheter and a hydrophilic guidewire to try to cross occlusion /during manipulation, guidewire entered the subintimal space of the CIA/	Temporary sealing with an angioplasty balloon./then insertion of a stent graft./expansion to 7 mm of the stent graft and the memotherm stent/no extravasation in the final angiogram. / Stable during the whole procedure. / Discharged the day after/ Without any medical problem in one‐month follow‐up	65‐year‐old female	Percutaneous recanalization of left common iliac artery	
4. U. Nyman et al.^19^		Balloon tamponade and then a stent graft were introduced/during the procedure, the patient became hypotensive. After closure, she was stabilized but, due to heart failure and cardiogenic shock, died 2 days later	A 69‐year‐old female	Valve replacement due to aortic valve stenosis. Then, intra atrial balloon pump insertion	Left common femoral artery
5. Francesco Paolo Busardò et al.^21^		Urgent laparotomy/evacuation of large retroperitoneal hematoma/sutured and use of metal clips/arterial ligation/2 h later, the patient's condition became critical, and they died shortly after	52‐year‐old man	Elective lumbar discectomy for a rightward disk herniation in the L4–L5 intervertebral space/	Left common iliac artery (10 mm tear of the left common iliac artery)
6. A. Chatziioannou et al.^22^		Massive acute extravasation/immediately covered with a stent, mounted on a balloon catheter/no extravasation was detected in the angiogram and was transferred to the intensive care unit/died 5 days later due to multiple organ failure	–	Percutaneous transluminal coronary angioplasty (PTCA)	Left common iliac artery perforation
7. Sabah Siddiqui et al.^20^	A 7 French (Fr) sheath was inserted/ Successful PCI deployment of an 8 Fr Angio‐Seal to the right femoral artery to achieve hemostasis after preclosure	Using balloon for lesion/subsequently covered with a covered stent/extravasation stopped/in 2 days later CT scan: Mild decrease in size in the right‐sided retroperitoneal, right pelvic sidewall, and right groin hematoma/transient worsening of chronic kidney disease/return of the renal function to baseline	76‐year‐old female	Left heart catheterization and interventional treatment for NSTEMI	Right external iliac artery extravasation
8. Vijay Trehan et al.^23^	Inserting 7F sheath in the right femoral artery by a modified seldinger technique/inserting 45‐cm (0.038‐in.) guidewire to the right femoral artery/difficulty in guidewire insertion	Unavailability of endovascular stent graft. One peripheral balloon catheter and the only peripheral stent graft available. The cranial end of the stent graft was hand‐crimped on the balloon, and the rest of the graft was crimped on the catheter shaft with the help of artery forceps, keeping the guidewire inside the lumen of the balloon catheter/transseptal sheath was deployed across the lesion passing of the assembled stent‐graft within a sheath and positioned at the site of the rupture/no extraluminal extravasation of the dye/hemodynamically stable after the procedure/normal flow without any leakage/discharged 3 days post‐procedure/	45‐year‐old diabetic male	Coronary artery angiography	Right external iliac artery
9. Varun Marimuthur et al.^24^	The Judkins right (JR) guide catheter was exchanged for an extra backup (EBU) catheter	Left FA was accessed, and a catheter/a peripheral balloon inflated across the EIA, covering the site of perforation/persistent leak and hemodynamic deterioration despite inflation for 5 cycles/simultaneous blood transfusion/the perforation was away from the origin of the Right IIA➔/a self‐made covered stent was prepared/sleeve of balloon material was removed./this balloon material was slid and mounted over a balloon expandable, rapid exchange stent. Two sutures were tied to the proximal and distal ends of the balloon material and the stent./covered stent deployed the sutures break/sandwich the balloon material between the vessel wall and the deployed stent. The sutures were given out, /angiogram and DSA were repeated/successful sealing good stent position in follow‐up CT/discharged. She was doing well at 6 months of follow‐up	70‐year‐old female	Coronary artery angiography	External iliac artery (EIA) with extravasation of blood
10. Chung‐Pei Chang et al.^25^	–	Repaired successfully/received 20 units of whole blood, 12 units of platelets, 10 units of frozen plasma, and 3000 mL of crystalloid fluid./extensive retroperitoneal hematoma/the leakage of contrast medium from the left internal iliac artery was noted./left hospital with good recovery in 1 week	A 29‐year‐old woman	Herniated left L5‐S1 intervertebral disc/microendoscopic discectomy	Tears of both left internal iliac artery and iliac vein

## AUTHOR CONTRIBUTIONS

**Maryam Mehrpooya:** Investigation; methodology; project administration; validation; writing – review and editing. **Massoud Ghasemi:** Project administration; supervision. **Pouya Ebrahimi:** Conceptualization; formal analysis; methodology; software; writing – original draft; writing – review and editing. **Homa Taheri:** Formal analysis; validation; writing – review and editing. **Parnian Soltani:** Conceptualization; data curation; resources; software.

## FUNDING INFORMATION

No funds have been received for this study.

## CONFLICT OF INTEREST STATEMENT

Authors declared no conflict of interest.

## CONSENT

Written informed consent was obtained from the patient for this study based on the patient's consent journal's policy.

## Data Availability

Further data will be provided by the corresponding author if there is a reasonable request.
